# FACTORS INFLUENCING HEALTH BEHAVIORS OF KOREAN OLDER ADULTS WITH CORONARY ARTERY DISEASE

**DOI:** 10.1093/geroni/igz038.3103

**Published:** 2019-11-08

**Authors:** So Young Shin, Ji Mi Mun

**Affiliations:** 1 Inje University, Busan, Korea, Republic of; 2 Busan Veterans Hospital, Busan, Korea, Republic of

## Abstract

Purpose: The purpose of this study was to examine the effects of disease-related knowledge, depression, and family support on health behaviors of older patients with coronary artery disease. Methods: The subjects were 139 older patients with coronary artery disease who had visited the internal medicine outpatient clinic at one general hospital located in metropolitan city B, Korea. A set of self-reported questionnaire was administered to assess general characteristics, disease-related knowledge, depression, family support, and health behaviors of the subjects. Collected data was analyzed using descriptive statistics, t-tests, one-way ANOVA, Pearson correlation coefficients, and multiple regression. Results: The mean (±SD) age of the subjects was 70.86 (±4.70) years. Health behaviors of the subjects had significant negative correlations with disease-related knowledge (r=-.17, p=.050) and depression (r=-.32, p<.001) while having a significant positive correlation with family support (r=.67, p<.001). In the final multiple regression analysis, factors influencing health behaviors of subjects were medication intake status (β=-.17, p=.009), depression (β=-.15, p=.017) and family support (β=.61, p<.001). The explanatory power of the subjects’ medication intake status, disease-related knowledge, depression and family support on health behaviors was 48.9% (F=33.97, p<.001). Conclusion: Medication intake status, depression, and family support had significant influences on health behaviors of older patients with coronary artery disease. Improvements in medication intake, depression, and family support for older patients with coronary artery disease may be beneficial for their health behaviors, and ultimately, have a positive effect on their recovery from the disease and well-being.

